# A long non-coding RNA *LncSync* regulates mouse cardiomyocyte homeostasis and cardiac hypertrophy through coordination of miRNA actions

**DOI:** 10.1093/procel/pwac019

**Published:** 2022-05-28

**Authors:** Rujin Huang, Jinyang Liu, Xi Chen, Ying Zhi, Shuangyuan Ding, Jia Ming, Yulin Li, Yangming Wang, Jie Na

**Affiliations:** Center for Stem Cell Biology and Regenerative Medicine, School of Medicine, Tsinghua University, Beijing 100084, China; School of Life Sciences, Tsinghua University, Beijing 100084, China; Center for Stem Cell Biology and Regenerative Medicine, School of Medicine, Tsinghua University, Beijing 100084, China; Center for Stem Cell Biology and Regenerative Medicine, School of Medicine, Tsinghua University, Beijing 100084, China; Capital Medical University, Beijing 100084, China; Center for Stem Cell Biology and Regenerative Medicine, School of Medicine, Tsinghua University, Beijing 100084, China; Center for Stem Cell Biology and Regenerative Medicine, School of Medicine, Tsinghua University, Beijing 100084, China; Capital Medical University, Beijing 100084, China; Beijing Key Laboratory of Cardiometabolic Molecular Medicine, Peking-Tsinghua Center for Life Sciences, Institute of Molecular Medicine, Peking University, Beijing 100871, China; Center for Stem Cell Biology and Regenerative Medicine, School of Medicine, Tsinghua University, Beijing 100084, China

**Dear Editor**,

Maintenance of cardiomyocyte (CM) homeostasis is essential for normal heart function. Long-term imbalance in heart homeostasis could elicit irreversible adaptive change in cell structure and function and tissue architecture, exemplified as cardiac hypertrophy and fibrosis, and eventually develop into heart failure (Shiojima et al., 2005). Therefore, identifying new genes and pathways regulating CM homeostasis may help better understand the cause of cardiac hypertrophy.

In recent years, researchers identified numerous long non-coding RNAs (lncRNAs) specifically expressed in the heart. However, many cardiac-specific lncRNAs seemed to be dispensable for heart development (Han et al., 2018; George et al., 2019). *LncSync* (*C430049B03Rik*) is a lncRNA located on mouse chrX:53053112–53057190 (^−^strand, mm10) and evolutionarily conserved in placental mammals (Fig. 1A). It has four exons, and exon 4 contains sequences encoding the miR-351 cluster: miR-351, miR-503, miR-322, and the target site of miR-181 (Fig. 1A). From E9.5 to E13.5, *LncSync* is significantly higher expressed in the heart than in other organs. In adult mice, it is only detected in the heart (Fig. 1B). RNA *in situ* hybridization revealed that *LncSync* is already detectable in the E9.5 heart and increased substantially in the ventricle at E10.5 and E11.5 (Fig. 1C). The expression pattern of *LncSync* can also be confirmed by the single-cell RNA-seq (scRNA-seq) analysis. It was expressed in *Tnnt2*^+^ embryonic CMs (Fig. S1A–D) (de Soysa et al., 2019; Pijuan-Sala et al., 2019). Similarly, *MIR503HG*, the ortholog of *LncSync* in humans, is enriched in the CMs of the human heart (Fig. S2A–D) (Haniffa et al., 2021). Subcellular fractionation assay showed that 75% of *LncSync* transcripts are present in the nucleoplasm and 23% in the cytoplasm, while *Xist* transcript was nearly 100% in the chromatin fraction (Fig. S3A). This result suggested that *LncSync* is a pri-microRNA transcript in the nucleoplasm. Accordingly, the expression levels of miR-351, miR-503, and miR-322 were parallel with that of *LncSync* during embryonic heart development (Fig. 1D). Intriguingly, *LncSync* also contained a sequence that is highly complementary to the seed sequence of the miR-181 family on exon 4 (Figs. 1E and S3B). We cloned the wild-type (WT) and mutated miR-181 targeting region of *LncSync* into a dual-luciferase reporter vector psiCheck-2 downstream of the Renilla luciferase (Fig. 1E). As expected, miR-181 significantly decreased the luciferase activity of the WT *LncSync* reporter compared to that of the mutant reporters (Fig. 1F). Transfection of miR-181 mimics to mESC overexpressing *LncSync* lead to a significant decrease in the levels of miR-351, miR-503, and miR-322 (Fig. S3C and 3D). Introducing miR-181 into purified E13.5 CMs also reduced *LncSync* and the miR-351 cluster levels (Fig. 1G). RNA-seq of E13.5 CMs transfected with miR-181 showed that 595 genes were significantly downregulated by miR-181. Gene Ontology (GO) terms associated with these genes include cardiac muscle growth, cardiac hypertrophy, and response to calcium ion (Figs. 1H and S3E), suggesting that miR-181 might impact CM development and function.

**Figure 1.**
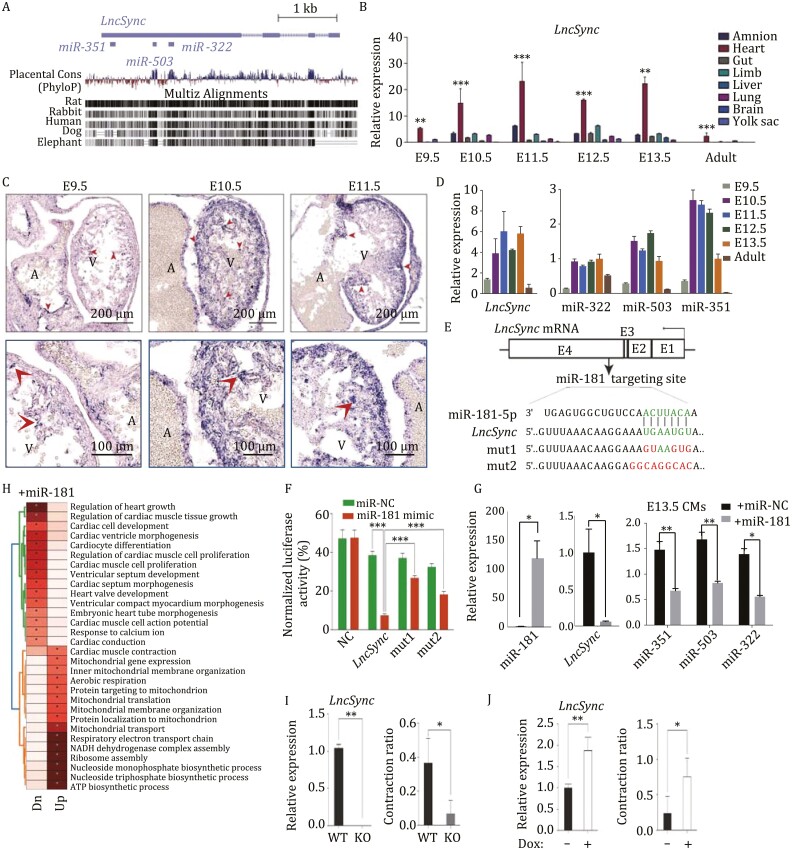
*LncSync* is an evolutionarily conserved LncRNA highly expressed in the embryonic heart. (A) Comparison of the genomic region of *LncSync* in placental mammals computed by PhyloP score. Predicted conserved sites are assigned positive scores (blue), fast-evolving sites are assigned negative scores (red). The multiz alignments were the probability that each nucleotide belongs to a conserved element based on the multiple alignments; sites predicted to be conserved were assigned positive scores (shown in black). (B) Q-PCR bar graph showing *LncSync* is highly enriched in the embryonic heart. *n* = 3 from three biological replicates. (Data represented as mean ± s.e.m., **P < 0.01, ***P < 0.001, based on unpaired Student’s *t*-test.) (C) RNA *in situ* hybridization of *LncSync* in mouse embryonic heart. A and V represent the atrium and ventricle, respectively. Red arrows indicate endocardium, myocardium, and epicardium in each subplot. Scale bar, 200 μm. (D) Q-PCR bar graph showing the expression of *LncSync* (left) and miR-351 cluster (right) during mouse heart development. *n* = 3 from three biological replicates. (Data represented as mean ± s.e.m.) (E) Schematic representation of the targeting site of miR-181-5p on *LncSync*. Mutated sites on “seed sequence” were labeled in red. (F) The bar graph of the luciferase reporter assay. Reporters contained the putative miR-181c target site in *LncSync* or mutant sequences. Luciferase reporter activity was normalized by firefly luciferase activity. *n* = 3 from three biological replicates. (Data represented as mean ± s.e.m., ***P < 0.001, based on unpaired Student’s *t*-test.) (G) Q-PCR bar graph showing the levels of miR-351, miR-503, miR-322 after transfection of miR-181 mimic (+miR-181) or control mimic (+miR-NC) in E13.5 CMs. *n* = 3. (Data represented as mean ± s.e.m., **P < 0.01, based on unpaired Student’s *t*-test.) (H) GO enrichment analysis for the genes upregulated (Up) or downregulated (Dn) after transfection of miR-181 mimic in E13.5 CMs. *n* = 2 independent batches of samples. (I) Q-PCR bar graph showing LncSync levels in WT and *LncSync*^−/−^ mESCs (left). Bar graph quantification of EB contraction ratio of *LncSync*^−/−^ and WT mESCs (right). *n* = 3 from three biological replicates. (Data represented as mean ± s.e.m., **P < 0.01, based on unpaired Student’s *t*-test.) (G) Q-PCR bar graph showing the level of *LncSync* in D10 EBs after Dox treatment (left). Bar graph quantification of EB contraction ratio after inducing *LncSync* expression (right). *n* = 3 from three biological replicates. (Data represented as mean ± s.e.m., **P < 0.01, based on unpaired Student’s *t*-test.)

During mESC cardiac differentiation, *LncSync* expression was highly upregulated in plated embryonic bodies (EBs) on day 10 (D10) (Fig. S3F and S3G). Knocking out *LncSync* in mESCs caused a marked decrease in the contraction ratio of *LncSyn*^*−*/*−*^ EBs (Fig. 1I). In the meantime, induced overexpression of *LncSync* using doxycycline (dox) -inducible system in α-MHC-GFP reporter mESC significantly increased the GFP fluorescence and the ratio of beating EBs on D10 (Figs. 1J, S3F and S3H). These data demonstrated that *LncSync* could promote CM differentiation *in vitro*.

We generated *LncSync*^*−*/*−*^ mouse by CRISPR (Figs. 2A and S4A). *LncSync*^*−*/*−*^ mice were born with the expected Mendelian ratio based on the genotyping result of 114 pups (Fig. S4B) and viable and fertile. The heart morphology, weight, and structure of E13.5–E17.5 *LncSync*^*−*/*−*^ and WT embryos did not show obvious abnormality (Fig. S4C and S4D). Interestingly, in 24 weeks old mouse, *LncSync*^*−*/*−*^ hearts became grossly larger, and the left ventricular wall appeared substantially thicker on the cross-section (Fig. 2B). The heart weight/tibia length ratio (HW/TL) of WT hearts is about 0.1 g/cm, while the HW/TL of *LncSync*^*−*/*−*^ hearts increased to 0.15 g/cm, almost 50% heavier (Fig. 2C). Moreover, echocardiography results revealed that the left ventricular mass (LVmass) increased significantly in 24 weeks old *LncSync*^*−*/*−*^ mice (Fig. 2D and 2E). Wheat Germ Agglutinin (WGA) staining showed that the cross-sectional area of *LncSync*^*−*/*−*^ CMs was 70% larger, indicating that the size but not the number of CMs increased after *LncSync* deletion (Fig. 2F and 2G). Masson’s trichrome staining of cross sections of the heart revealed that areas with collagen deposition in *LncSync*^*−*/*−*^ hearts reached about 4%, significantly more extensive than that in the WT heart (about 1%) (Fig. 2H and 2I). In addition to the apparent cardiac hypertrophy and fibrosis, *LncSync*^*−*/*−*^ mice also developed hypertension. We measured the blood pressure of 24 weeks *LncSync*^*−*/*−*^ mice and found their blood pressure significantly higher than the WT mice (Fig. S5A). The morphology of the capillaries in the renal corpuscle and the lung appeared normal (Fig. S5B), suggesting that the *LncSync*^*−*/*−*^ mouse did not have pathological changes in the blood vessel. To prove that the cardiac hypertrophy of *LncSync*^*−*/*−*^ mice was not due to high blood pressure, we gave 13-weeks WT and *LncSync*^*−*/*−*^ mice angiotensin II (Ang II) for 28 days. The SBP and LV mass of *LncSync*^*−*/*−*^ mice were already significantly higher than that of WT mice at 13 weeks, but the difference narrowed over time, and we did not observe a significant difference between these two groups by 17 weeks (Fig. S5C, S5D, S5G and S5H). After 28 days, Ang II caused a significant increase in LV area in 17 weeks WT heart (Fig. S5E and S5F). On the other hand, untreated *LncSync*^*−*/*−*^ hearts displayed hypertrophy compared to WT hearts at 17 weeks, and there was little difference in *LncSync*^*−*/*−*^ hearts with or without Ang II treatment (Fig. S5E and Fig. S5E). These results strongly suggest that *LncSync*^*−*/*−*^ hearts became hypertrophic in a cell-autonomous manner and responded poorly to Ang II. Next, we purified E13.5 and 8 weeks old adult CMs and performed high-throughput sequencing of miRNAs and mRNAs (Figs. 2A and S6A). Both WT and *LncSync*^*−*/*−*^ CMs highly expressed CMs markers (*Tnnt2, Tnni3*), but lowly expressed EC markers (*Kdr*), smooth muscle cell (SMC) markers (*Tagln*), hematopoietic stem cell (HSC) markers (*Ly6a, Kit*), and pluripotent stem cell (PSC) markers (*Nanog*, *Sox2*, *Pou5f1*) (Fig. S6B). Eight hundred and fifty two and 324 genes were significantly up- and downregulated in the E13.5 *LncSync*^*−*/*−*^ CMs, respectively (Fig. 2J). GO and KEGG analysis revealed a significant enrichment of genes related to cardiac hypertrophy and ferroptosis (a form of regulated cell death caused by lipid hydroperoxides) in the *LncSync*^*−*/*−*^ CMs (Figs. 2K and S6C), suggesting that the deletion of *LncSync* induced a certain degree of ferroptosis in the embryonic heart. Similarly, 8-weeks adult *LncSync*^*−*/*−*^ CMs also significantly upregulated hypertrophic genes: α-skeleton actin 1 (*Acta1*), brain natriuretic peptide β (*Nppb*), and regulator of calcineurin 1 (*Rcan1*) and fibrotic genes: Collagen1a1 (*Col1a1*) and Collagen3a1 (*Col3a1*) (Fig. S6D).

**Figure 2.**
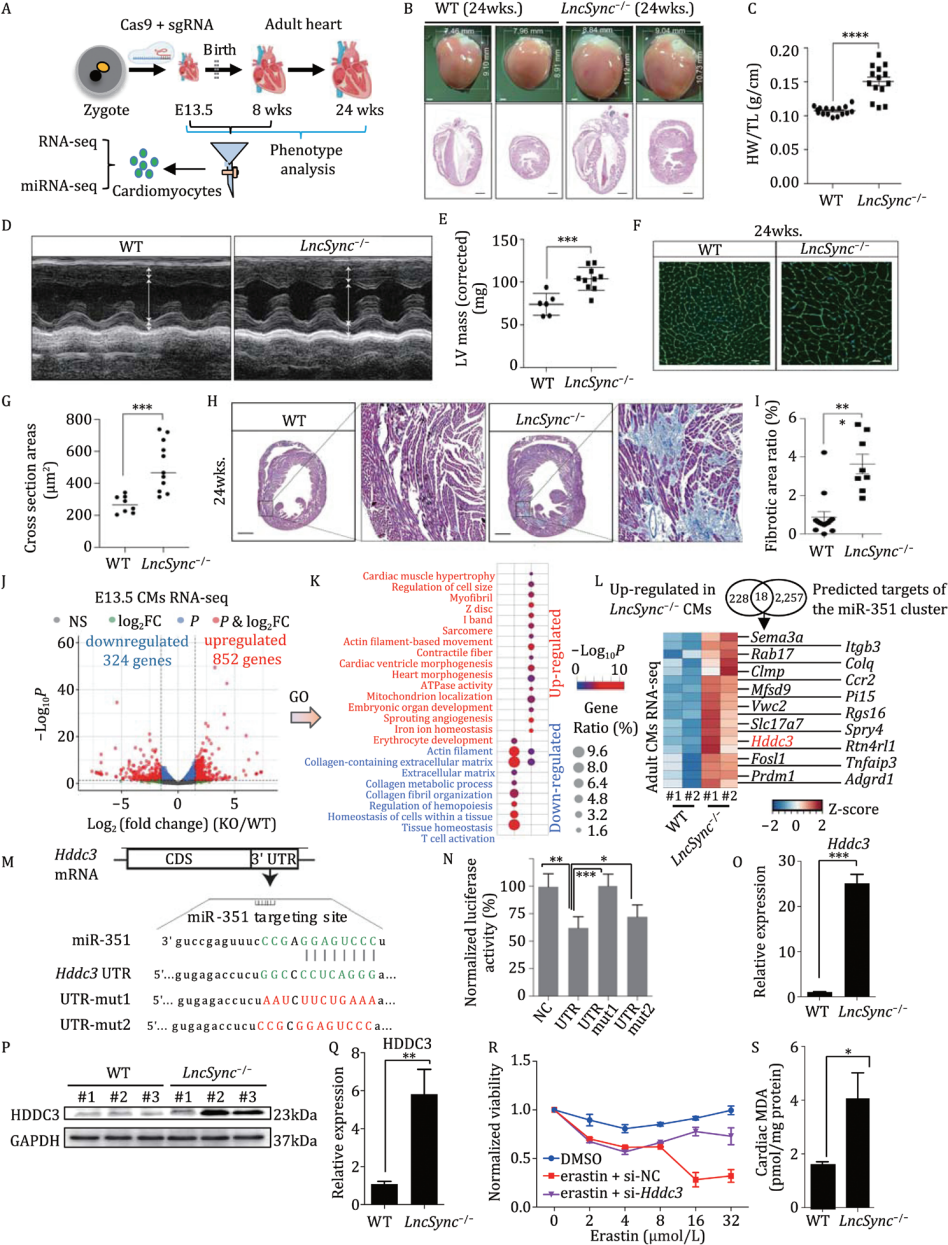
*LncSync* ^−/−^ mouse developed pathological cardiac hypertrophy. (A) Schematic diagram of *LncSync*^−/−^ mice generation and phenotype analysis. (B) Gross appearance and HE staining of *LncSync*^−/−^ and WT mouse hearts (24 weeks). Scale bar in the lower subplot, 1 mm. (C) Heart weight (HW)/tibia length (TL) ratio of 24 weeks old mouse. *n* = 16 for the WT group and *n* = 14 for *LncSync*^−/−^ group. (Data represented as mean ± s.e.m., ****P < 0.0001, based on unpaired Student’s *t*-test.) (D) M-mode echocardiographic images of *LncSync*^−/−^ and WT adult heart (24 weeks). (E) Analysis of LVmass (Left Ventricular mass [corrected] [mg]) of hearts (24 weeks). *n* = 6 for the WT group and *n* = 10 for *LncSync*^−/−^ group. (Data represented as mean ± s.e.m., ***P < 0.001, based on unpaired Student’s *t*-test.) (F) WGA-stained sections to measure cardiomyocyte transverse diameters in heart tissue from *LncSync*^−/−^ mouseversus WT animals. Scale bar, 20 μm. (G) Quantification of CM size in WT and *LncSync*^−/−^ hearts. *n* = 8 for the WT group and *n* = 11 for *LncSync*^−/−^ group. (Data represented as mean ± s.e.m., ***P < 0.001, based on unpaired Student’s *t*-test.) (H) Masson’s trichrome staining reveals the fibrotic area in WT and *LncSync*^−/−^ heart sections. The collagen deposition in the fibrotic area appeared blue, and normal CMs appeared pink. Scale bar, 1 mm. (I) Quantification of the fibrotic area, *n* = 10 for the WT group and *n* = 8 for *LncSync*^−/−^ group. (Data represented as mean ± s.e.m., ***P < 0.001, based on unpaired Student’s *t*-test.) (J) Volcano plot of differentially expressed genes in WT and *LncSync*^−/−^ E13.5 CMs. Red-colored dots indicate genes with fold change ≥ 1.5 and *P* ≤ 0.05. *n* = 2 independent batches of samples. (K) GO analysis for genes significantly upregulated (Up, terms labeled in red) and downregulated (Dn, terms labeled in blue) in the *LncSync*^−/−^ E13.5 CMs. (L) Venn diagram indicating the number of candidate genes upregulated in the *LncSync*^−/−^ CMs and the predicted target genes of the miR-351 cluster (top). Heatmap showing the expression of overlapped 18 candidate genes (bottom). (M) Schematic representation of the miR-351 targeting site in *Hddc3* 3ʹUTR. WT and mutated “seed sequence” are colored in green and red, respectively. (N) Bar graph showing the normalized luciferase activity of reporters with the WT or mutated miR-351-5p putative target sites in *Hddc3*. Luciferase reporter activity was normalized by Renilla luciferase activity. *n* = 3 from three biological replicates. (Data represented as mean ± s.e.m., *P < 0.05, **P < 0.01, ***P < 0.001, based on unpaired Student’s *t*-test.) (O) Q-PCR bar graph showing *Hddc3* levels in WT and *LncSync*^−/−^ adult CMs. *n* = 3 from three biological replicates. (Data represented as mean ± s.e.m., ***P < 0.001, based on unpaired Student’s *t*-test.) (P) Western blot showing HDDC3 protein expression in WT and *LncSync*^−/−^ hearts (8 weeks). (Q) Quantification of HDDC3 protein expression level in (P). *n* = 3 from three biological replicates. (Data represented as mean ± s.e.m., **P < 0.01, based on unpaired Student’s *t*-test.) (R) The relative viability of E13.5 CMs in response to erastin treatment after transfection of 500 nmol/L control siRNA (si-NC) or *Hddc3*-siRNA (si-*Hddc3*) for 48 h. Erastin concentrations are indicated. DMSO, labeled in blue. *n* = 3 from three biological replicates. (Data represented as mean ± s.e.m., *P < 0.05, based on unpaired Student’s *t*-test.) (S) Malondialdehyde (MDA) levels in WT and *LncSync*^−/−^ hearts (8 weeks). Values were normalized with protein level. *n* = 3 from three biological replicates. (Data represented as mean ± s.e.m., *P < 0.05, based on unpaired Student’s *t*-test.)

We analyzed the miRNA profile of WT and *LncSync*^*−*/*−*^ CMs. As expected, *LncSync* hosted miR-351 cluster, including passenger strands (miR-503-3p, miR-351-3p, miR-322-3p), were the only microRNAs massively downregulated in both embryonic and adult *LncSync*^*−*/*−*^ CMs (Fig. S6E and S6F). To investigate the downstream target of *LncSync*-derived miRNAs *in vivo*, we performed miRNA target analysis using target prediction databases MirDB (Chen and Wang, 2020), Miranda (Betel et al., 2008), and TargetScan (Agarwal et al., 2015). Eighteen genes were significantly upregulated in the *LncSync*^*−*/*−*^ CMs and predicted targets of the miR-351 cluster (Fig. 2L). *Hddc3* is a protein-coding gene whose expression level increased substantially in both embryonic and adult *LncSync*^*−*/*−*^ CMs (Fig. 2L). Sequence analysis and luciferase reporter assay confirmed that *Hddc3* is a direct target of miR-351 (Fig. 2M and 2N). The RNA and protein levels of *Hddc3* raised significantly in the *LncSync*^*−*/*−*^ heart (Figs. 2O–Q and S7A). These results strongly suggest that *Hddc3* is upregulated in *LncSync*^*−*/*−*^ heart as a target of miR-351. A recent study reported that human *HDDC3* (also named *MESH1*) is a cytosolic NADPH phosphatase, and the depletion of *HDDC3* could protect cells from ferroptosis (Ding et al., 2020). To test whether *Hddc3* can regulate ferroptosis in CMs, we treated E13.5 CMs with ferroptosis inducer erastin. Compared to the DMSO group, erastin treatment reduced CM viability to 0.3 (Fig. 2R). When *Hddc3* was knocked down with siRNA (Fig. S7B), cell viability remained 0.7–0.9 with erastin treatment (Fig. 2R). This result indicated that reducing *Hddc3* levels could indeed protect CMs from ferroptosis. Moreover, RNA-seq revealed that markers indicative of ferroptosis (Yang et al., 2014; Fang et al., 2019, 2020), such as *Ptgs2*, *Tfrc* (transferrin receptor), *Slc7a11,* and *Fth1* (ferritin heavy polypeptide 1), were significantly upregulated in the *LncSync*^*−*/*−*^ CMs (Fig. S7B). The ferroptosis end-product malondialdehyde (MDA) elevated markedly in *LncSync*^*−*/*−*^ hearts (Fig. 2S). Transmission electron microscopy revealed that mitochondria were distorted and shrunken in the 24 weeks old *LncSync*^−/−^ adult CMs (Fig. S7C). The above results suggest that in the *LncSync*^−/−^ heart, the absence of the miR-351 cluster caused the derepression of *Hddc3,* which led to dysregulation of CMs metabolism and activation of the ferroptosis pathway.

Our study revealed the important physiological function of *LncSync* in mammalian hearts and uncovered a delicate cross-regulation pattern between lncRNA and microRNAs. *LncSync* and miR-351 control the levels of *Hddc3*. Without *LncSync*, HDDC3 protein levels increase, which might disrupt the metabolic balance in CMs and makes them susceptible to ferroptosis, and the KO mice eventually developed pathological cardiac hypertrophy. The regulatory relationship between *LncSync* and miR-181 is also interesting. There have been many reports about the versatile roles of miR-181 family members in embryo development and physiological and pathological processes, particularly in the cardiovascular system (Sun et al., 2014). We showed that miR-181 could target *LncSync* hence its derived miR-351 cluster. This finding expanded the scope of miR-181 targets. According to our microRNA profiling, the miR-181 level is significantly higher in adult CMs than in embryonic CMs (Fig. S6G and S6H), which may downregulate the *LncSync* post-transcriptionally and its derived miR-351 cluster in adult mice. The growth, differentiation, and function of the CMs need constant adjusting depending on the developmental stage and the physiological requirement. *LncSync* and the miR-351 cluster might serve as a rheostat to regulate CMs fate specification and homeostasis, for example, by reducing *Hddc3* levels. Besides, *LncSync* could be under the control of miR-181 to fine-tune the CMs’ status in response to specific conditions. Deleting *LncSyn*c abrogates such regulation and eventually leads to dysregulation of CM homeostasis, slow onset of ferroptosis, and hypertrophic remodeling of the heart.

## Supplementary Material

pwac019_suppl_Supplementary_Material_S1

pwac019_suppl_Supplementary_Material_S2

## Footnotes

This work was supported by the National Natural Science Foundation of China (NSFC) (Grant Nos. 91740115 and 31970819), the National Key R&D Program of China (Grant Nos. 2017YFA0102802 and 2019YFA0110001), the funding from Tsinghua-Peking Center for Life Sciences and core facilities of Tsinghua-Peking Center for Life Sciences.

Rujin Huang, Jinyang Liu, Xi Chen, Ying Zhi, Shuangyuan Ding, Jia Ming, Yulin Li, Yangming Wang, and Jie Na declare they have no conflict of interest.

This article does not contain any studies with human subjects performed by any of the authors. All institutional and national guidelines for the care and use of laboratory animals were followed. The laboratory animal facility has been accredited by AAALAC (Association for Assessment and Accreditation of Laboratory Animal Care International), and the IACUC (Institutional Animal Care and Use Committee) of Tsinghua University approved all animal protocols used in this study.

All data generated or analyzed during this study are included in this published article (and its supplementary information files). The RNA and microRNA high-throughput sequencing data are publicly available at the National Centre for Biotechnology Information with Gene Expression Omnibus with accession number GSE146032.

RH and JN conceived, planned the study, and wrote the manuscript. RH performed the experiments and obtained most of the data. JL, XC, YZ, SD, JM participated in the data collection, YL and YW helped with experimental design, and all authors approved the manuscript. We thank Daren Wu, Fan Jiang, Wenxue Gu, Yanzhu Yue, and Shanshan Ai for their assistance. We appreciate Drs Bailong Xiao, Cecilia Canessa, and Aibin He for the discussion and constructive suggestions. All authors read and approved the final version of the manuscript.
