# Supplementary material for: A long non-coding RNA LncSync regulates mouse cardiomyocyte homeostasis and cardiac hypertrophy through coordination of miRNA actions
Source: Protein Cell. 2022 May 28;14(2):153–7. doi: 10.1093/procel/pwac019 (PMC10019570; doi:10.1093/procel/pwac019)
Supplement: pwac019_suppl_Supplementary_Material_S1 [file pwac019_suppl_supplementary_material_s1.docx]

**A long non-coding RNA *LncSync* regulates mouse cardiomyocyte homeostasis and cardiac hypertrophy through coordination of miRNA actions**

**Summary**

Long non-coding RNAs (lncRNAs) have been reported to play various regulatory roles in cardiogenesis *in vitro*, while their *in vivo* physiological functions remain elusive. Here we show that a heart enriched lncRNA, *LncSync*, is required to maintain the heart homeostasis. *LncSync* is the host of the miR-351 cluster, and it can be targeted by miR-181. Deletion of *LncSync* impairs mESCs cardiac differentiation *in vitro*. The addition of miR-181 downregulates the miR-351 cluster expression and affects cardiomyocytes (CMs) differentiation program. *In vivo*, *LncSync^-/-^* CMs significantly upregulated genes associated with hypertrophic cardiomyopathy and ferroptosis. *LncSync* deletion causes a substantial increase in *Hddc3* transcripts and protein. *Hddc3* is a target gene of miR-351 and a facilitator of ferroptosis. Adult *LncSync^-/-^* mice developed pathological cardiac hypertrophy with increased CMs size and heart fibrosis. Our study identified a novel lncRNA-miRNA regulation pattern that fine-tunes CMs homeostasis and shed insight on the physiological function of non-coding RNAs in the mouse heart.

**Supplemental Figure Legends**

**
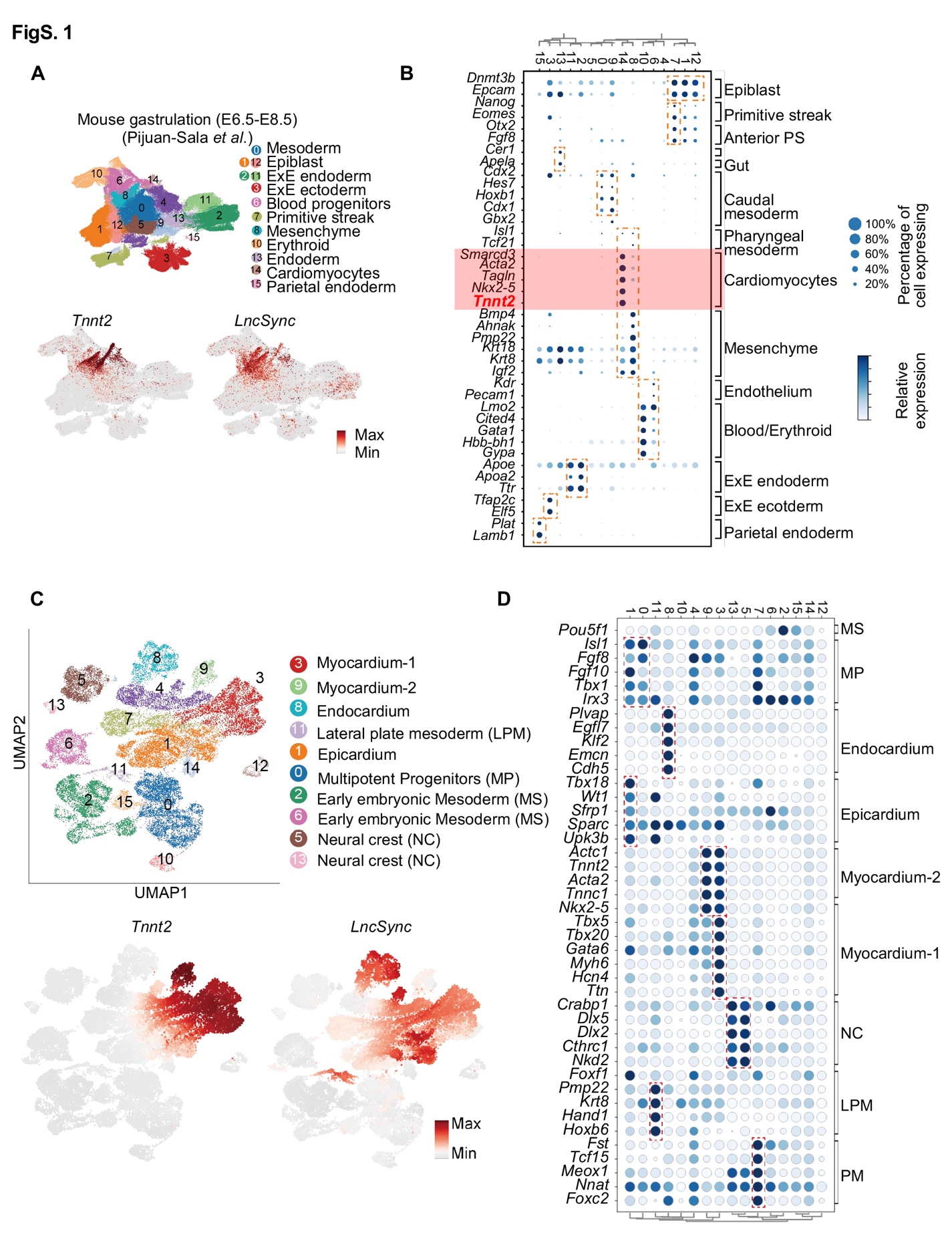
**

**Fig. S1 | *LncSync* is highly expressed in the embryonic heart.**

1. Single-cell RNA-seq analysis of *LncSync* expression in the E6.5-E8.5 mouse embryos. Top: E6.5-8.5 mouse embryonic cell clusters segregation by UMAP identified 13 different cell types. Bottom: UMAP plots show *LncSync*+ cells largely overlap with *Tnnt2*+ cells (scaled from low-gray to high-red).
2. Dot plot showing expression of a selection of marker genes for the indicated cell lineages in the scRNA-seq dataset from E6.5-E8.5 mouse embryos. Dot size indicates the percentage of cells expressing a specific gene within the indicated cluster; the color indicates the average expression level (scaled from low-white to high-blue).
3. Single-cell RNA-seq re-analysis of LncSync expression during cardiogenesis in E7.75-E9.25 mouse embryos. Top: embryonic cardiac cell clusters segregation by UMAP identified 15 different cell types. Bottom: UMAP plots showed *LncSync*^+^ cells belonging to the myocardium, endocardium, and *Tnnt2*^+^ (scaled from low-gray to high-red).
4. Dot plot representing the expression of marker genes of the indicated cell lineages in the scRNA-seq dataset from E7.75-E9.25 mouse hearts. The dot size reflects the percentage of cells expressing a specific gene; the color indicates the average expression level (scaled from low-white to high-blue).


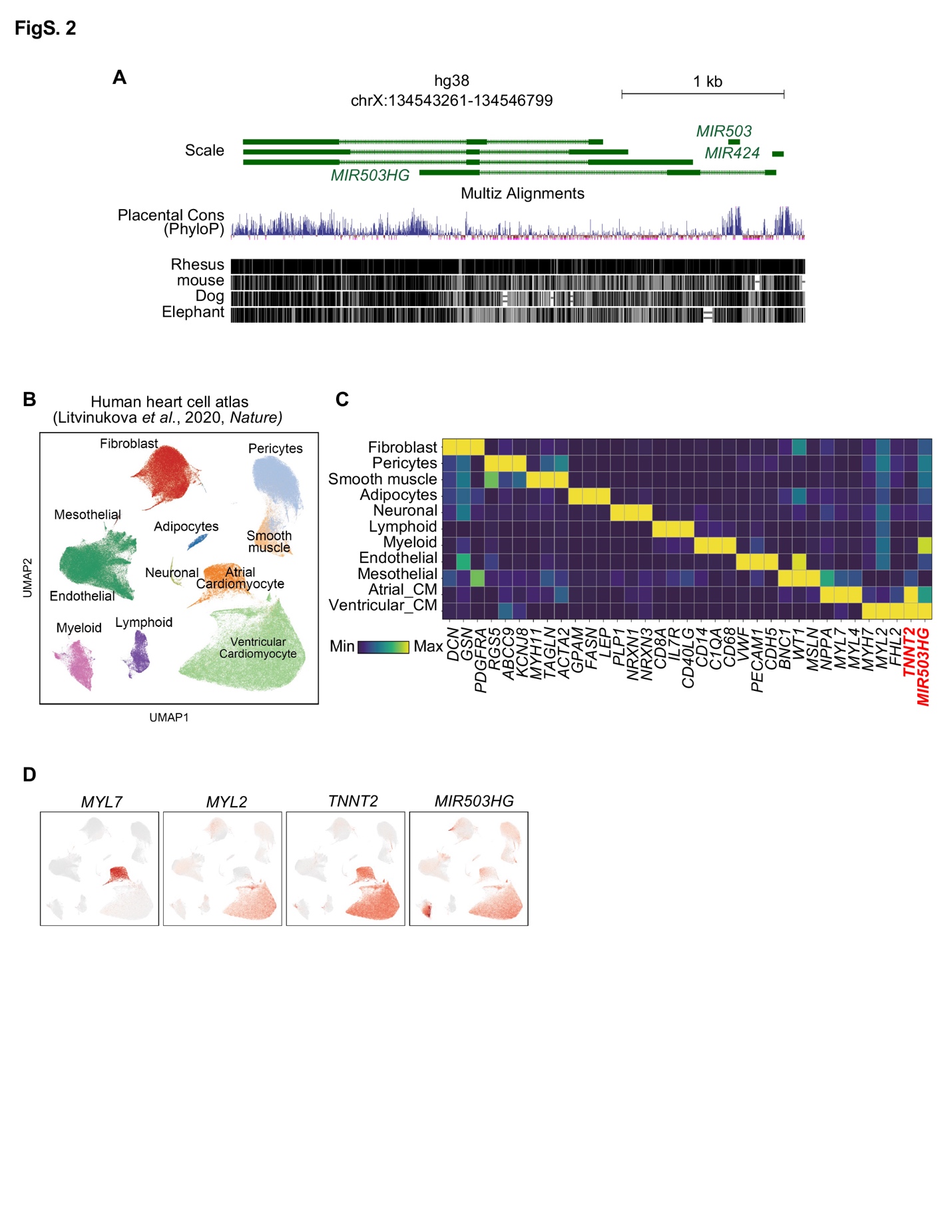


**Fig. S2 | *MIR503HG* is specifically enriched in the CMs of the human heart.**

1. The genomic region of *MIR503HG* in humans.
2. Uniform manifold approximation and projection (UMAP) embedding of 487,106 human heart cells. UMAP layout and cell-type annotation were from the original study.
3. Expression of marker genes for panel (**B**), marker genes were from the original study.
4. UMAP shows the expression of selected marker genes and *MIR503HG*. Note that *MIR503HG* is specifically expressed in ventricular cardiomyocytes.


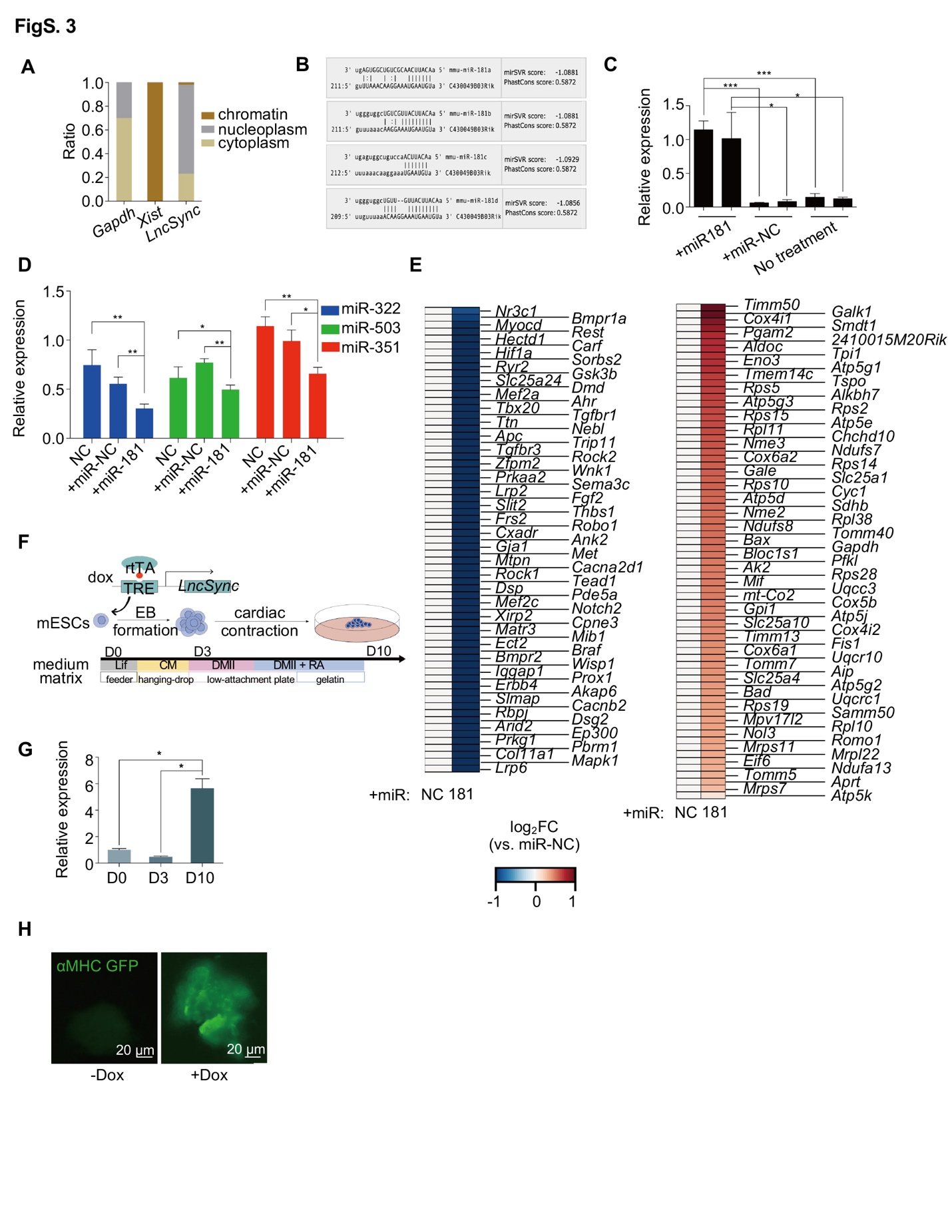


**Fig. S3 | *LncSync* was processed into the miR-351 cluster and promoted mESC cardiac differentiation *in vitro*.**

1. Q-PCR measured the relative expression level of *Gapdh* (cytoplasm control), *Xist* (chromatin control), and *LncSync* at different subcellular localization. Note that *LncSync* is mostly in nucleoplasm and cytoplasm.
2. MiR-181a, b, c, d/LncSync alignment details. MirSVR is a machine learning program to rank microRNA target sites by a downregulation score. The PhastCons score is a probability that each nucleotide belongs to a conserved element, where positive values imply conservation.
3. Q-PCR bar graph showing miR-181 levels after transfection of 10 nM (+miR-181-1) or 20 nM (+miR-181-2) miR-181 or control mimics ((+miR-NC1 & 2). N = 2 from 2 biological replicates. (Data represented as mean ± S.E.M., ns, not significant, *P < 0.05, ***P < 0.001, based on unpaired Student’s t-test).
4. Q-PCR bar graph showing the relative expression level of the miR-351 cluster after miR-181 mimic transfection during mESCs cardiac differentiation. mESCs were transfected with miR- 181 mimic (+miR-181) or control mimics (+miR-NC), NC represented the non-transfected control group. Values were normalized against non-transfected mESCs on D4 of differentiation. N = 3 from 3 biological replicates. (Data represented as mean ± S.E.M., *P < 0.05, **P < 0.01, based on unpaired Student’s t-test).
5. Heatmap showing representative differentially expressed genes in E13.5 heart CMs after transfection of miR-181 mimic (+miR-181) or control mimic (+miR-NC) in embryonic CMs. N = 2 independent batches of samples.
6. Schematic view of the doxycycline-inducible *LncSync* overexpression system during mESC cardiac differentiation.
7. Q-PCR bar graph showing the expression of *LncSync* during mESCs cardiac differentiation from D0 to D10. N = 3 (Data represented as mean ± S.E.M., *P < 0.05, based on unpaired Student's t-test).
8. Induced *LncSync* overexpression activated αMHC-GFP reporter. Scale bar, 20 μm.


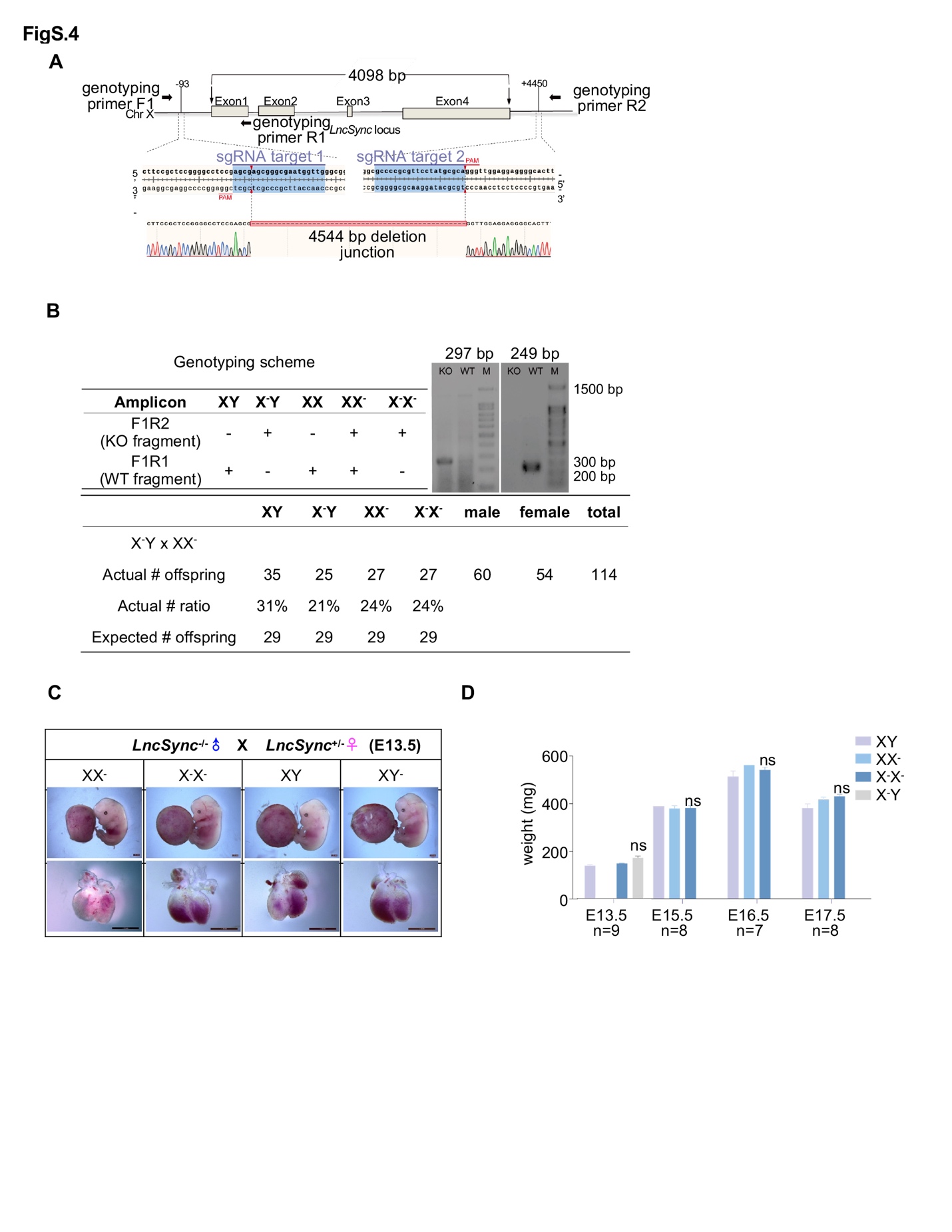


**Fig. S4 | The *LncSync*^-/-^ mouse did not show obvious abnormality at the embryonic stages.**

1. sgRNAs targeting the 5' and 3' end of the *LncSync* gene (position and sequence shown) were designed. Target sequences (blue shade), PAMs (pink underline), Cas9 cleavage sites (red triangles). A 4544 bp deletion confirmed by Sanger sequencing is shown below.
2. Genotyping scheme of *LncSync*^-/-^, heterozygote (Het), and WT mouse. The table at the bottom shows *LncSync*^-/-^ mice offspring ratios and the expected Mendelian ratio.
3. Images of *LncSync*^-/-^ and WT E13.5 embryos, placentas, and heart. Scale bar, 1 mm.
4. The bodyweight of *LncSync*^-/-^, *LncSync*^+/-^, and WT embryos at indicated stages. N = 7-9, from multiple batches of embryos. Data represented as mean ± S.E.M. ns, not significant, based on unpaired Student's t-test.


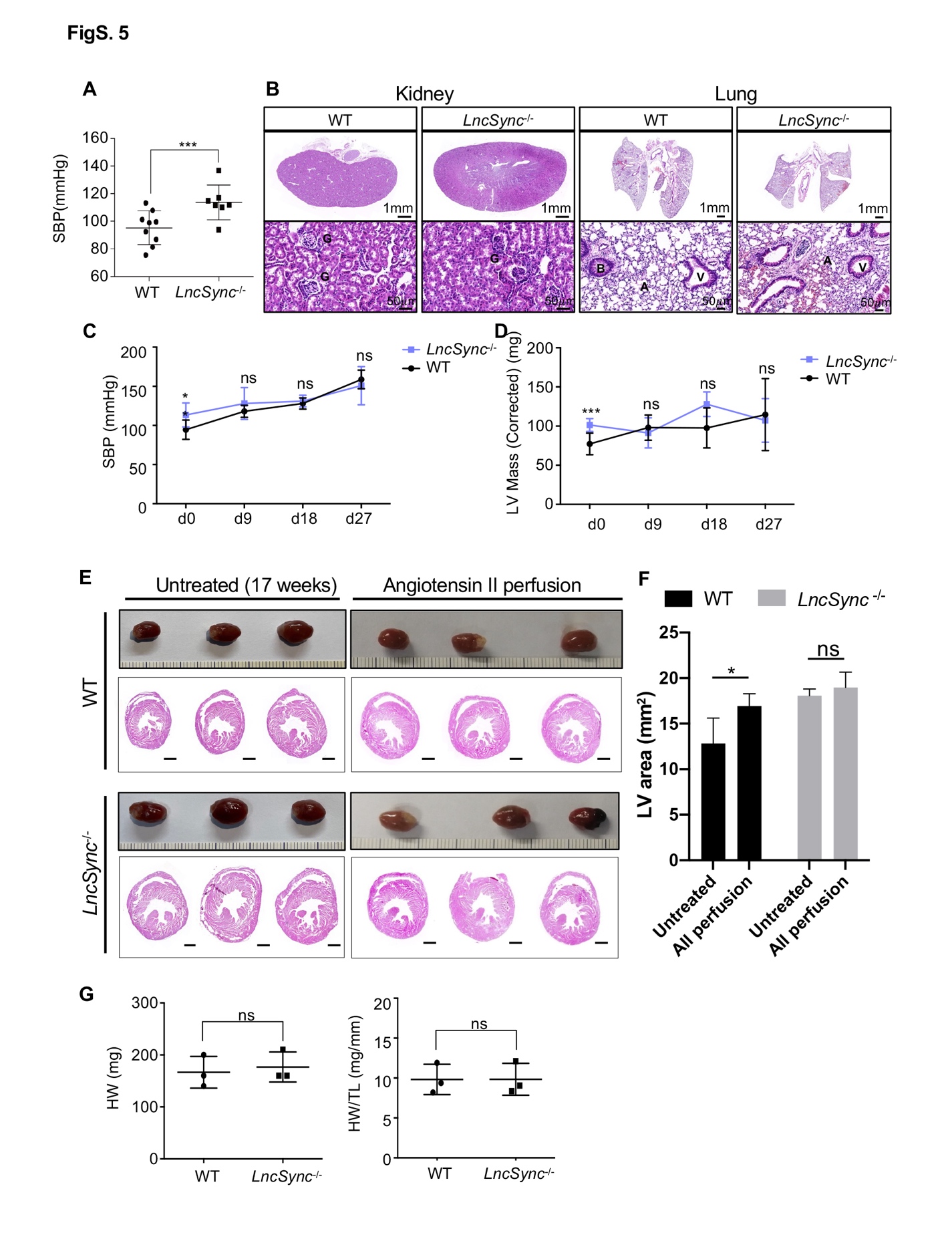


**Fig. S5 | Phenotype analysis of *LncSync^-/-^* mouse.**

1. Systolic blood pressure (SBP) of *LncSync^-/-^* and WT 24-weeks mice. N = 9 for the WT group and N=7 for *LncSync^-/-^* group. (Data represented as mean ± S.E.M., ***P < 0.001, based on unpaired Student’s t-test).
2. Histological analysis of the kidney (left) and lung (right) of *LncSync*^-/-^ and WT adult mice (24 wks.). G, glomerulus. Scale bar as indicated.
3. Systolic blood pressure (SBP) of *LncSync*^-/-^ and WT adult mice during angiotensin II (Ang II) infusion (start from 13 wks.). N = 3 from 3 biological replicates. (Data represented as mean ± S.E.M., **P < 0.01, ns, not significant, based on unpaired Student’s t-test).
4. Analysis of LVmass (Left Ventricular mass (corrected) (mg)) of *LncSync*^-/-^ and WT adult mouse hearts during Ang II infusion (start from 13 wks.). N = 3 from 3 biological replicates. (Data represented as mean ± S.E.M., ***P < 0.001, ns, not significant, based on unpaired Student’s t-test).
5. Gross appearance and HE staining of paraffin sections of *LncSync*^-/-^ and WT mouse (17 wks.) hearts after 28-day-Ang II infusion. Scale bar, 1 mm.
6. Quantification of the short axial section area of left ventricle in WT and *LncSync*^-/-^ hearts. N=3 for each group. (Data represented as mean ± S.E.M., *P < 0.05, based on unpaired Student’s t-test).
7. Heart weight (HW) (left) and HW/tibia length (TL) (right) ratio of 24 wks. mice. N = 16 for the WT group and N=14 for *LncSync^-/-^* group. (Data represented as mean ± S.E.M., ns, not significant, based on unpaired Student’s t-test).


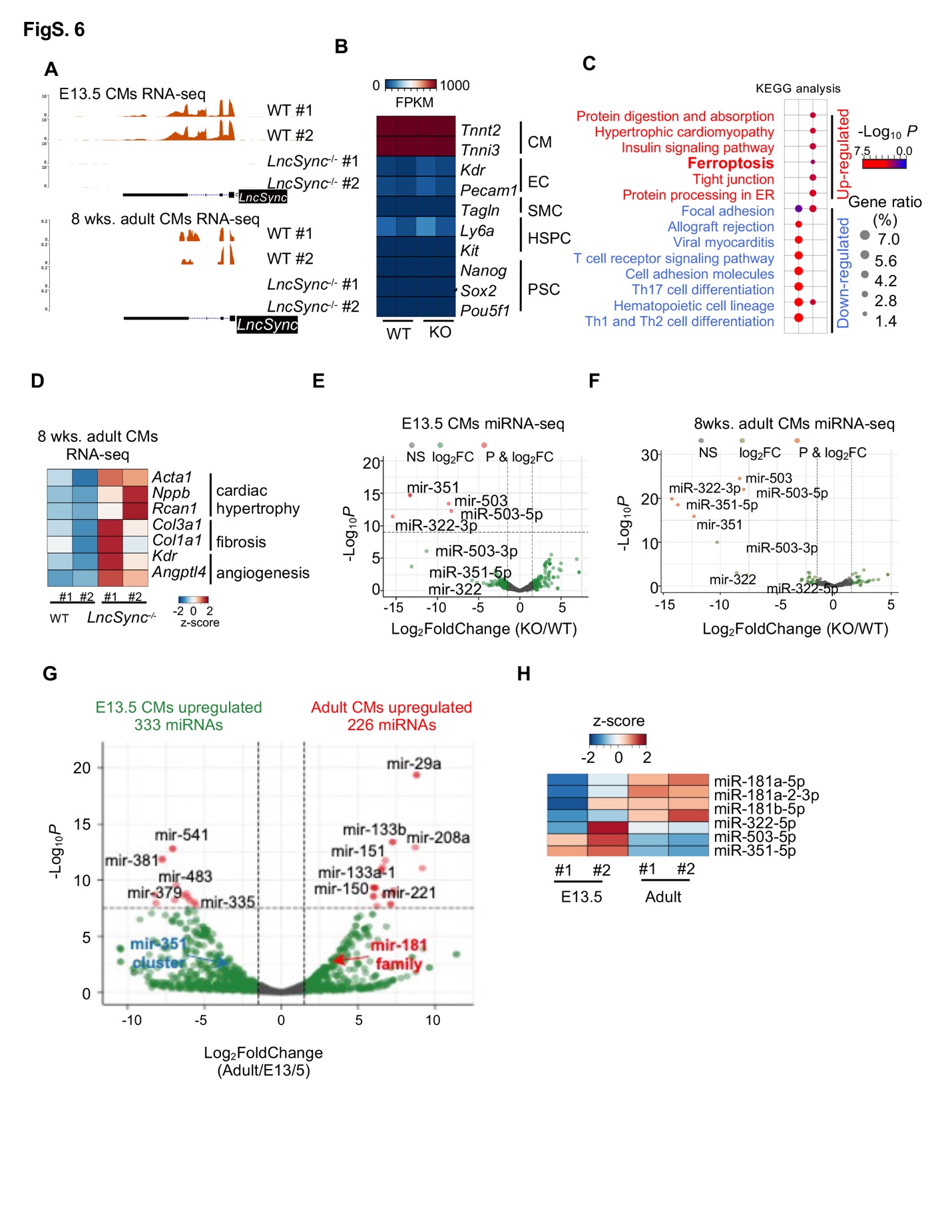


**Fig. S6 | Transcriptome profiling revealed hypertrophic alteration in *LncSync*^-/-^ embryonic and adult mouse CMs.**

1. *LncSync* transcript detected by RNA-seq in WT and *LncSync*^-/-^ E13.5 and adult CMs.
2. Heatmap showing purified CMs highly expressed CM marker genes and lowly expressed non-CM genes for both WT and *LncSync*^-/-^ groups.
3. Kyoto encyclopedia of genes and genomes (KEGG) analysis of genes significantly upregulated (red) and downregulated (blue) in the *LncSync*^-/-^ E13.5 CMs.
4. Heatmap shows the expression level of selected genes associated with cardiac hypertrophy, fibrosis, angiogenesis, and cardio-protection in adult CMs.
5. Volcano plot of differentially expressed miRNAs between WT and *LncSync*^-/-^ E13.5 CMs. Red dots indicate genes with FoldChange ≥ 1.5 and P ≤ 10^-9^.
6. Volcano plot of differentially expressed miRNAs between WT and *LncSync*^-/-^ adult CMs. Red dots indicate genes with FoldChange ≥ 1.5 and P ≤ 10^-16^.
7. Volcano plot of differential expressed miRNAs between E13.5 and adult WT mouse CMs. Red-colored dots indicate genes with FoldChange ≥ 1.5 and P ≤ 10^-7^.
8. Heatmap showing the expression of miR-181 family and the miR-351 cluster in embryonic and adult mouse CMs.


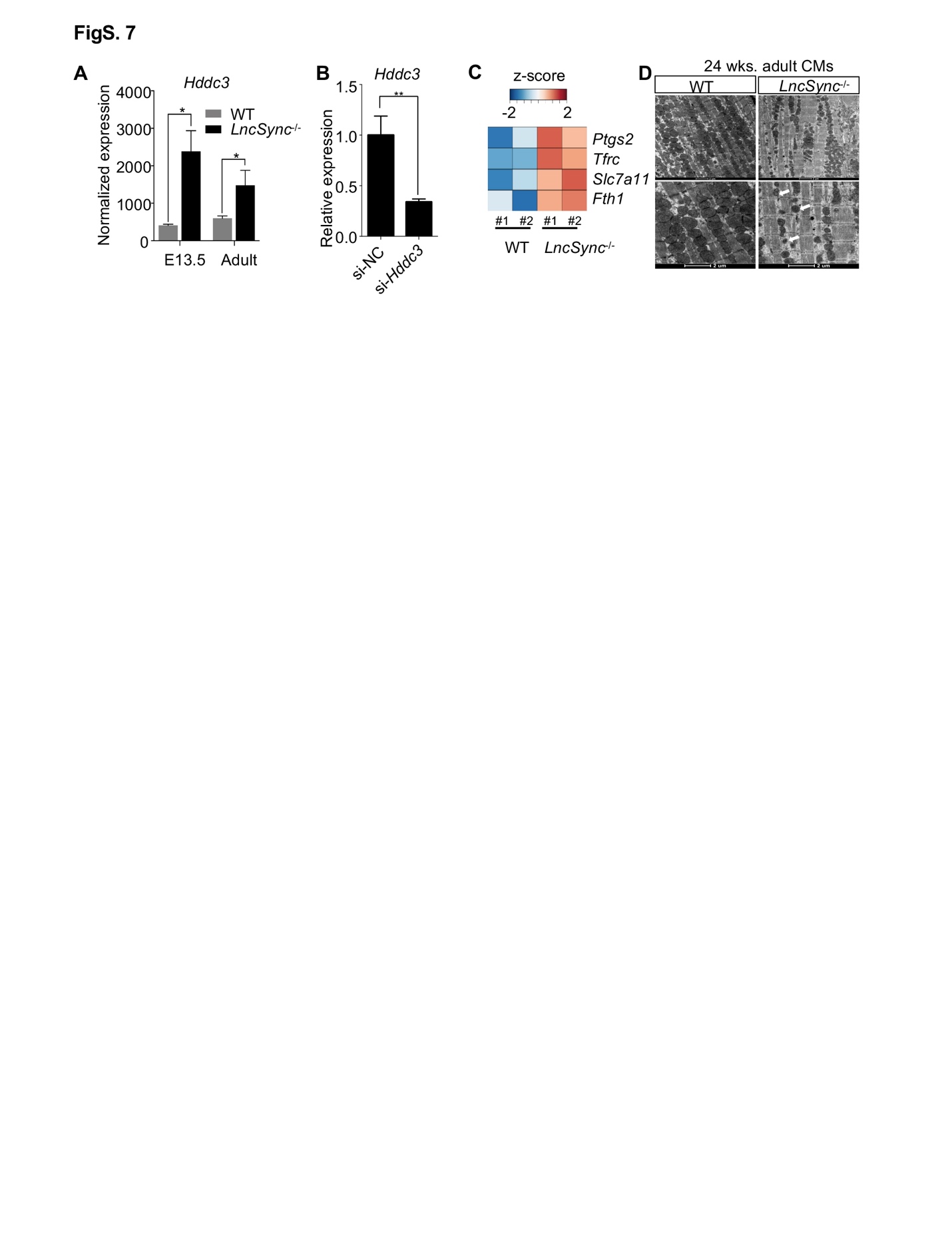


**Fig. S7 |Analysis of *Hddc3* expression and ferroptosis in *LncSync*^-/-^ CMs**

1. Bar graph showing the expression level of *Hddc3* after the loss of *LncSync* in E13.5 and adult CMs based on RNA-seq (Data represented as mean ± S.E.M., *P < 0.05, based on unpaired Student's t-test).
2. Q-PCR bar graph showing the expression level of *Hddc3* after transfection *Hddc3* siRNA (si-Hddc3) or control siRNA (si-NC). N = 3 from 3 biological replicates. (Data represented as mean ± S.E.M., **P < 0.01, based on unpaired Student's t-test).
3. Heatmap showing the change for ferroptosis-related marker gene expression in WT and *LncSync*^-/-^ CMs (8 wks.).
4. Transmission electron microscopy (TEM) analysis of WT and *LncSync*^-/-^ CMs (24 weeks old). White arrowheads, shrunken mitochondria. A minimum of 5 fields per condition was examined.

**Supplemental Tables**

**Table S1.**

The sequence of primers used for q-PCR, genotyping and plasmid construction, and the sequence of sgRNA, miRNA mimics used in this study.

**Supplemental Methods**

***LncSync^-/-^* mouse generation**

All institutional and national guidelines for the care and use of laboratory animals were followed. Two sgRNAs targeting the full-length *LncSync* were designed using the CRISPR DESIGN website (http://crispr.mit.edu/) and inserted into the pUC57 vector. The DNA fragments containing T7 promoter-driven sgRNAs were amplified for *in vitro* transcription using MEGAshortscript T7 Transcription Kit (Invitrogen Cat #AM1354). 10 ng/μL sgRNAs and 50 ng/μL Cas9 mRNA were injected into zygotes from C57BL/6J mouse (RRID: IMSR_JAX:000664). Injected zygotes were transferred into pseudo-pregnant C57BL/6J female mice. The genomic DNA from the offspring was extracted and verified by PCR; the primers and sgRNA sequences are listed in **Table S1**.

**Heart section and histology analysis**

Mouse embryonic (E8.5 to E17.5) and adult hearts were dissected, washed with cold PBS, and fixed in paraformaldehyde (PFA) overnight at 4℃. The samples were dehydrated using an automation-tissue-dehydrating machine (LEICA ASP200S) and embedded in paraffin. The tissues and organs used in this study included the limb, heart, liver, brain, intestine, lung, yolk sac, umbilical cord, and placenta, which were carefully separated with fine forceps under a dissection microscope.

For hematoxylin and eosin (H&E) staining, Masson's staining, and WGA staining, 5 μm sections were deparaffinized in xylene and rehydrated through 100%, 95%, 75%, 50% ethanol, then stained for H&E, Masson's Trichrome or WGA-FITC (Invitrogen Cat #L4895). For CM size quantification, representative WGA-FITC images were calculated using ImageJ.

**RNA *in situ* hybridization**

The hearts were dissected from E8.5 to E13.5 embryos from pregnant mice before fixing overnight in 4% PFA at 4°C. After fixation, they were rinsed in 1×DEPC PBS, immersed in 1×DEPC-PBS contained 20~30% sucrose (Invitrogen, CAS 57-50-1, Cat #N15503-022), and embedded in OCT (Leica, Cat #14020108926) and frozen sectioned (18~20 μm thick).

DIG-labeled antisense and sense RNA probes were transcribed *in vitro* using MEGAscript T7 Transcription Kit (Invitrogen Cat #AM1334). For RNA *in situ* hybridization, slides were washed twice and treated with 10 μg/mL proteinase K in PBS for 20 min, then postfixed in 4% PFA in PBS for 20 min. After rinsing with PBS, the slides were prehybridized in the fresh hybridization buffer for 1 hour at room temperature (RT), followed by incubation in hybridization buffer and 0.1 ng/mL digoxin (DIG)-labeled *LncSync* probe overnight at 65 °C. On the next day, slides were washed with prewarmed 0.2×SSC buffer for 4 times, then rinsed in Buffer B1 for 5 min twice before blocking with goat serum in RT for 1 hour, and replate with fresh blocking reagent containing 1:5,000 dilution of alkaline phosphatase (AP)-anti-DIG antibody (Roche, Cat#11093274910, RRID: AB_514497), and incubated at 4 °C overnight. Afterward, slides were washed with Buffer B1 three times and equilibrated in Buffer B3 at RT. Finally, the slides were incubated with AP substrate at RT for desired time then stopped with ddH_2_O.

**Plasmid construction, mouse ESC transfection, and CM differentiation**

*LncSync* cDNA was cloned into pLenti-EF1α to generate a constitutive *LncSync* OE mESCs. For inducible expression of *LncSync*, the cDNA product was cloned into FU-tet-o-hOct4 (Addgene, Cat #19778, RRID: Addgene_19778) or pPB-TRE3G (a gift from Sanger Institute). To knock out *LncSync* in mESC, we used the sgRNA design tool from MIT (<http://crispr.mit.edu/>) to design 4 sgRNAs flanking the 5' and 3'-end of full-length *LncSync*. They were cloned individually into the Bsa I site in the pUC57-T7-sgRNA plasmid (**Table S1**).

For CM differentiation, mESCs were cultured in 20 µl hanging droplets of mESCs culture medium without LIF to form EBs. Three days later, we harvest EBs, culture them in the Petri dishes for another 1.5 days and treat with Bmp4 (0.4 ng/mL), Activin A (10 ng/mL), VEGF (5 ng/mL), and Ascorbic Acid (1mM). Afterward, EBs were plated separately into gelatin (0.1%, Corning, Cat #3599)-coated 96-well plates. The medium was changed every two days. The morphology and beating behaviour of EBs were monitored by light microscopy at 37°C on day 10. GFP^+^ cells could be observed in beating clusters in R1-αMHC-GFP^+^ reporter lines three days after plating. The contraction ratio is calculated as “The number of beating EBs” divided by “Total number of EBs”.

**RNA extraction and qPCR**

Total RNA was extracted from the cells using Trizol reagent (Invitrogen, Cat #15596018). One μg of total RNA was used to synthesize cDNA with the 5× All-In-One RT MasterMix (Abm, Cat #G490). Produced cDNA was diluted in water, and 16.7 ng was used in each qPCR reaction. qPCR was performed using GoTaq qPCR Master Mix (Promega, Cat #A6002) in a CFX96 Real-Time System (Bio-Rad), and results were analyzed with the Bio-Rad CFX Manager program. The Beta-Actin gene was used as an internal control for the qPCR analysis of gene expression. Each experiment was performed in triplicate and repeated three times. The primer sequences used in this study are listed in **Table S1**.

**Bulk RNA-seq and bioinformatic analysis**

Two hundred ng total RNA was reverse transcribed and amplified using Smart-seq2^13^. Two independent biological replicates of each group were sequenced using Illumina HiSeq2500. Clean reads were aligned to the GENCODE annotations transcriptome (M14) using Bowtie2 (version 2.2.5) and RSEM (version 2.4.1, RRID:SCR_013027). Differentially expressed genes were obtained using DESeq2 (version 1.10.1) with adjusted p-value < 0.05 and fold change > 2 as the threshold. Cluster analysis of gene expression patterns was performed using Glbase3^14^. GO analysis was performed using an R package ClusterProfiler (version 3.10.1).

For miRNA profiling, miRNA high-throughput sequencing was performed by HiSeq2500, SE50, producing over 10 million reads from each sample. Clean reads were mapped to the mouse genome (mm10) using miRDeep2^15^.

**Single-cell RNA-seq data analysis**

Fastq reads were aligned to the genome using STARsolo^16^ with the setting '--outSAMattributes NH HI AS nM CR CY UR UY --readFilesCommand zcat --outFilterMultimapNmax 100 --winAnchorMultimapNmax 100 --outMultimapperOrder Random --runRNGseed 777 --outSAMmultNmax 1'. The count matrix was lightly filtered to exclude cell barcodes with low numbers of counts: Cells with less than 1000 UMIs, less than 500 genes, or more than 20% fraction of mitochondrial counts were removed. The filtered matrix was normalized using scran^17^. The top 4000 most highly variable genes were used for PCA, and the first 50 PCs (principal components) were used for downstream analysis with SCANPY. MAGIC was used to denoise and impute gene expression to overcome the dropout effect in single-cell data.

**Echocardiography and blood pressure measurement**

Echocardiography was performed using the VisualSonics Vevo 2100 system equipped with a 40 Mhz 550 s probe. Mice of different ages (3, 5, 8, 10, 13, and 24 wks.) were placed on a platform with integrated physiologic monitoring capabilities. M-mode images were acquired from awake mouse and used to calculate fractional shortening. Systolic and diastolic blood pressure was measured daily in mice utilizing a tail-cuff placed on the tail to occlude the blood flow for one week.

**CM isolation from embryonic and adult mouse heart**

For embryonic CM isolation, E13.5 mouse hearts were removed and placed in ice-cold 1×HBSS. After dissection and rinsing with 1×PBS 3 times, the ventricles were cut into 0.5-1 mm^3^ fragments with scissors and digested overnight on a 4°C shaking table with 0.05% trypsin. On day 2, the tissue was put into a 50 mL centrifuge tube with collagenase II (Worthington, Cat #LS004176) (1 mg/mL) and digested for 10 min in a 37°C water bath. The supernatant with dissociated CMs was collected, and the above operation was repeated until the tissue block disappeared. After centrifuging the cell suspension at 25×g for 3 min, the cell mixture was plated onto a 10 cm tissue culture dish for 1 hour, and the non-adherent cells were mostly CMs.

For adult mouse heart CMs isolation, hearts were rapidly removed from anesthetized mice and mounted onto a temperature-controlled (37°C) Langendorff apparatus via the aorta. They were first perfused with a Ca^2+^-free perfusion solution for 2 min, then were digested with a perfusion buffer containing Collagenase II (0.9 mg/mL) and Protease (Sigma, Cat# P5147-1G) (0.06 mg/mL) for ~10 min until the tissue looked pale. The perfusion solution (pH 7.4) contained the following: NaCl 137 mM, KCl 5.4 mM, MgCl_2_ 1.2 mM, HEPES 20 mM, NaH_2_PO_4_ 1.2 mM, glucose 10 mM, taurine 10 mM, and the solution was gassed with 100% O^2^. The digested heart was then removed from the cannula, and the left ventricle was cut into small pieces in the perfusion solution. Centrifuge the cell suspension at 25×g for 1 min to pellet the CMs. At least 90% of viable rod-shaped CMs have clear striated sarcomere structures.

**Luciferase Assays**

Luciferase reporters were generated by cloning the fragment of the 3'UTR sequence (~200 bp) into the psiCheck-2 vector (A gift from Kehkooi Kee lab) or pmirGLO vector. Then 293FT cells were co-transfected with 100 ng of luciferase reporters and 100 nM miRNA mimics by Lipofectamine 2000 (Thermo Fisher, Cat# 11668019). Cell lysates were harvested 24 and 48 hours after transfection. Luciferase activities were measured using the Dual-Luciferase Reporter Assay System (Promega, Cat# E1910). Renilla luciferase values were normalized to firefly luciferase in the psiCheck-2 vector, and firefly luciferase values were normalized to renilla luciferase in the pmirGLO vector.

**Statistical analysis**

Experiments in this study were done from at least three biological repeats. Data were presented as mean ± S.E.M. calculated using GraphPad Prism. Statistical differences were determined by unpaired two-tailed Student's t-test. P-values < 0.05 was considered statistically significant. No samples were excluded from any analysis.

**Data availability**

The RNA and microRNA high-throughput sequencing data are publicly available at the National Centre for Biotechnology Information with Gene Expression Omnibus with accession number **GSE146032**. Data are accessible with a reviewer token: "inuriuqsnvarhcf".
